# Antidepressant effects of selective adenosine receptor antagonists targeting the A1 and A2A receptors administered jointly with NMDA receptor ligands: behavioral, biochemical and molecular investigations in mice

**DOI:** 10.1007/s43440-024-00627-z

**Published:** 2024-07-25

**Authors:** Aleksandra Szopa, Karolina Bogatko, Anna Serefko, Mariola Herbet, Marta Ostrowska-Leśko, Andrzej Wróbel, Maria Radziwoń-Zaleska, Jarosław Dudka, Piotr Wlaź, Ewa Poleszak

**Affiliations:** 1 Department of Clinical Pharmacy and Pharmaceutical Care, Medical University of Lublin, Chodźki 7, Lublin, PL 20–093 Poland; 2 Chair and Department of Applied and Social Pharmacy, Laboratory of Preclinical Testing, Medical University of Lublin, Chodźki 1, Lublin, PL 20–093 Poland; 3 Chair and Department of Toxicology, Medical University of Lublin, Chodźki 8, Lublin, PL 20–093 Poland; 4 Second Department of Gynecology, Medical University of Lublin, Jaczewskiego 8, Lublin, PL 20–090 Poland; 5 Department of Psychiatry, Medical University of Warsaw, Nowowiejska 27, Warszawa, PL 00–665 Poland; 6 Department of Animal Physiology and Pharmacology, Institute of Biological Sciences, Faculty of Biology and Biotechnology, Maria Curie-Skłodowska University, Akademicka 19, Lublin, PL 20–033 Poland

**Keywords:** DPCPX, Istradefylline, NMDA receptor ligands, Forced swim test, BDNF, Gene expression

## Abstract

**Background:**

The objective of the study was to ascertain the antidepressant potential of the co-administration of NMDA receptor ligands and selective adenosine A1 and A2A receptor antagonists.

**Methods:**

The forced swim test (FST) and spontaneous locomotor activity test were carried out in adult male naïve mice. Before the behavioral testing, animals received DPCPX (a selective adenosine A1 receptor antagonist, 1 mg/kg) or istradefylline (a selective adenosine A2A receptor antagonist, 0.5 mg/kg) in combination with L–701,324 (a potent NMDA receptor antagonist, 1 mg/kg), D–cycloserine (a partial agonist at the glycine recognition site of NMDA receptor, 2.5 mg/kg), CGP 37849 (a competitive NMDA receptor antagonist, 0.3 mg/kg) or MK–801 (a non-competitive NMDA receptor antagonist, 0.05 mg/kg). Additionally, serum BDNF level and the mRNA level of the *Adora1*, *Comt*, and *Slc6a15* genes in the murine prefrontal cortex were determined.

**Results:**

The obtained results showed that DPCPX and istradefylline administered jointly with NMDA receptor ligands (except for DPCPX + D–cycloserine combination) produced an antidepressant effect in the FST in mice without enhancement in spontaneous motility of animals. An elevation in BDNF concentration was noted in the D–cycloserine-treated group. *Adora1* expression increased with L–701,324, DPCPX + D–cycloserine, and DPCPX + CGP 37849, while D–cycloserine, CGP 37849, and MK–801 led to a decrease. *Comt* mRNA levels dropped with DPCPX + L–701,324, istradefylline + L–701,324/CGP 37849 but increased with D–cycloserine, MK–801, CGP 37849 and DPCPX + MK–801/ CGP 37849. *Slc6a15* levels were reduced by D–cycloserine, DPCPX + L–701,324 but rose with DPCPX + CGP 37849/MK–801 and istradefylline + D–cycloserine/MK–801/CGP 37849.

**Conclusion:**

Our study suggests that selective antagonists of adenosine receptors may enhance the antidepressant efficacy of NMDA receptor ligands highlighting a potential synergistic interaction between the adenosinergic and glutamatergic systems. Wherein, A2A receptor antagonists are seen as more promising candidates in this context. Given the intricate nature of changes in BDNF levels and the expression of *Adora1*, *Comt*, and *Slc6a15* seen after drug combinations exerting antidepressant properties, further research and integrative approaches are crucial understand better the mechanisms underlying their antidepressant action.

## Introduction

It is estimated that approximately one-quarter of the global population, or 280 million individuals, suffer from a depressive disorder [1]. Given the high prevalence and frequent misdiagnosis, the World Health Organization has identified this as a serious public health concern. It is a well-established phenomenon that antidepressant drugs commonly prescribed for the treatment of depression increase the levels of biogenic amines (noradrenaline, dopamine, serotonin) in the central nervous system (CNS) [2, 3]. These drugs help stabilize the mood of many patients and their daily functioning, but only one in three people using them showed significant improvement after two months of pharmacotherapy. Furthermore, taking antidepressants is associated with side effects (among others anticholinergic– dry mouth, constipation, blurred vision; gastrointestinal– nausea and vomiting, diarrhea or constipation, weight changes; cardiovascular– increased heart rate, orthostatic hypotension; neurological– headache, dizziness; sexual– decreased libido, erectile dysfunction, delayed ejaculation or anorgasmia), which are the most common cause of patient non-compliance and discontinuation of treatment [4, 5].

Scientific reports indicate that the glutamatergic system is implicated in the pathophysiology of depressive disorders [6–8]. Therefore, substances that modulate various sites in the *N*-methyl-D-aspartate (NMDA) receptor complex may represent a new therapeutic strategy for people suffering from depression [2]. For instance, D–cycloserine, a partial/full agonist of NMDA receptors is used with satisfactory outcomes in polytherapy of patients with treatment-resistant depression [9]. Similarly, amantadine, the non-selective NMDA receptor antagonist, led to significant clinical improvement, if it was used with a common antidepressant (imipramine) in polytherapy of drug-resistant unipolar depressed patients [10]. A review of preclinical and clinical studies indicates that ketamine and memantine, non-competitive antagonists of NMDA receptors, may be beneficial in the treatment of depression (for review see [11]). Moreover, esketamine– the *S*-enantiomer of ketamine, has recently received the Food and Drug Administration (FDA) approval for use as an intranasal drug delivery system (Spravato™) for treating resistant depression [12]. For many years, the potential of ionic ligands of the NMDA receptor, i.e., Mg^2+^ and Zn^2+^, in the prevention and treatment of depressive disorders has been evaluated. The outcomes obtained from both preclinical and clinical research provided compelling evidence that these ions possess antidepressant activity [13, 14] and their capacity to augment the antidepressant effects of frequently prescribed pharmaceuticals, such as imipramine, fluoxetine, citalopram, reboxetine, and paroxetine, which non-selectively or selectively inhibit the reuptake of noradrenaline, serotonin and/or dopamine, as well as, moclobemide acting as an inhibitor of their degradation, which contributes to enhancement of the level of these biogenic amines in the CNS [15–17].

A literature review reveals that a synergistic interaction has been described between the adenosinergic and glutamatergic systems in relation to several disease states [18–25]. Nevertheless, the precise role of this interplay in the etiology and treatment of affective disorders, such as depression, remains unclear. A notable observation was made regarding the activation of NMDA receptors, which has been shown to result in the release of adenosine in the striatum and cerebral cortex of rats. Furthermore, it has been established that the activation of adenosine receptors exerts a reduction in the NMDA receptor-mediated effects. [26, 27]. According to the literature, the glutamatergic system is strongly connected with the adenosinergic system. There are several possible mechanisms of their interaction: (1) adenosine through the stimulation of A1 and A2A receptors modulates the release of glutamate (Glu) [28], (2) adenosine by inhibiting the depolarization, elevates the threshold for the opening of the NMDA receptor ion channel, thus reducing NMDA receptor activation [18], (3) the interplay between NMDA and A1 receptors results in the decreased presynaptic release of Glu in different brain areas [29], and (4) the inhibition of NMDA receptors decreases extracellular levels of adenosine [30, 31]. However, except for Mg^2+^ and Zn^2+^ ions [32], no attempt has been made to study interactions between selective A1 and A2A adenosine receptor antagonists and ligands modulating various sites in the NMDA receptor complex, in the context of their antidepressant potential.

Thus, the primary objective of the present study was to evaluate the impact of selective A1 and A2A adenosine receptor antagonists on the antidepressant efficacy of NMDA receptor ligands in the forced swim test (FST) in mice. Additionally, the influence of the applied combinations on the serum concentration of brain-derived neurotrophic factor (BDNF) whose concentration is modified in the course of depression [33, 34], and on the expression of selected genes involved in neuronal signaling (*Adora1*, *Comt*, and *Slc6a15*) was evaluated.

## Materials and methods

### Pharmacological agents and chemical compounds

The following substances were used: CGP 37849 (dl–(E)–amino–4–methyl–5–phosphono–3–pentenoic acid, Abcam Biochemicals, Cambridge, UK; no. ab120127)– a competitive NMDA receptor antagonist, D–cycloserine (d–4–amino–3–isoxazolidone, Sigma–Aldrich, Poznań, Poland; no. 30,020)– a partial agonist at the glycine recognition site of NMDA receptor, DPCPX (8–cyclopentyl–1,3–dipropylxanthine, Sigma–Aldrich, Poznań, Poland; no. C101)– a selective adenosine A1 receptor antagonist, istradefylline ((E)–8–(3,4–dimethoxystyryl)–1,3–diethyl–7–methylxanthine, Sigma–Aldrich, Poznań, Poland; no. SML0422)– a selective adenosine A2A receptor antagonist, L–701,324 (7–chloro–4–hydroxy–3–(3–phenoxy)phenylquinolin–2[1 H]–one, Sigma–Aldrich, Poznań, Poland; no. L0258)– a potent NMDA receptor antagonist, and MK–801 (dizocilpine, Sigma–Aldrich, Poznań, Poland; no. M107)– a non-competitive NMDA receptor antagonist.

The following commercial kits and reagents were used for biochemical and molecular studies: enzyme-linked immunosorbent assay (ELISA) kit for BDNF (Cloud–Clone Corp., Katy, TX, USA; no. APA011Mu01), high-capacity cDNA reverse transcription kit (Applied Biosystems, Foster City, CA, USA; no. 4368814), gene-specific TaqMan probe (Applied Biosystems, Waltham, Massachusetts, USA; no. 4351368), RNAase–free water (EURx, Gdańsk, Poland; no. E0210), TRIzol Reagent (Invitrogen, Carlsbad, CA, USA; no. 15-596-026), Fast Probe qPCR Master (EURx, Gdańsk, Poland; no. E0422), and ROX Solution (EURx, Gdańsk, Poland; no. E0402).

The following chemicals were utilized in the experiment: chloroform (POCH, Gliwice, Poland; no. 234431116), ethanol (POCH, Gliwice, Poland; no. 396420113), isopropanol (POCH, Gliwice, Poland; no. 751500111).

### Laboratory animals

A total of 150 male albino Swiss mice, aged 10–12 weeks and weighing 25–30 g, were purchased from the Experimental Medicine Centre (Lublin, Poland) for use in the study. The animals were maintained in cages with standard dimensions and a capacity of 8 mice per cage. The environmental parameters within the room where the animals were housed were strictly controlled, with a temperature of 21 ± 1 °C, a relative humidity of 50 ± 5%, and a 12-h light/dark cycle. The animals were provided with unrestricted access to food and tap water. In accordance with the regulations set by Polish law to studies on animal models, all experimental procedures involving mice were conducted with the utmost care and attention to detail, adhering strictly to the guidelines established by the European Community Council (2010/63/EU). When planning animal research, the 3Rs (Replacement, Reduction, and Refinement) principle was considered. All protocols and experimental procedures involving mice were reviewed and approved by the Local Ethics Committee for Animal Testing in Lublin (license number 51/2019).

### Experimental procedures

A solution of CGP 37849 (0.3 mg/kg), D–cycloserine (2.5 mg/kg), and MK–801 (0.05 mg/kg) was prepared in saline, while a suspension of DPCPX (1 mg/kg), istradefylline (0.5 mg/kg), and L–701,324 (1 mg/kg) was prepared in saline with 1% Tween 80 (POCH, Gliwice, Poland). CGP 37849, D–cycloserine, L–701,324, and MK–801 were administered *intraperitoneally* (*ip*) 60 min, while DPCPX was administered *ip* 30 min before behavioral testing. Istradefylline was administered by oral gavage (*po*) 60 min before the experiment. The control group received two administrations of saline (0.9% NaCl), i.e. the first one *po* 60 min and the second *ip* 30 min before behavioral testing. Freshly prepared solutions and suspensions or saline were administered at volumes of 5 or 10 ml/kg for *po* or *ip* administration, respectively.

Drug administration schedule: (1) saline + saline, (2) saline + DPCPX, (3) saline + istradefylline, (4) L–701,324 + saline, (5) L–701,324 + DPCPX, (6) L–701,324 + istradefylline, (7) D–cycloserine + saline, (8) D–cycloserine + DPCPX, (9) D–cycloserine + istradefylline, (10) CGP 37849 + saline, (11) CGP 37849 + DPCPX, (12) CGP 37849 + istradefylline, (13) MK–801 + saline, (14) MK–801 + DPCPX, (15) MK–801 + istradefylline.

**Scheme 1:**
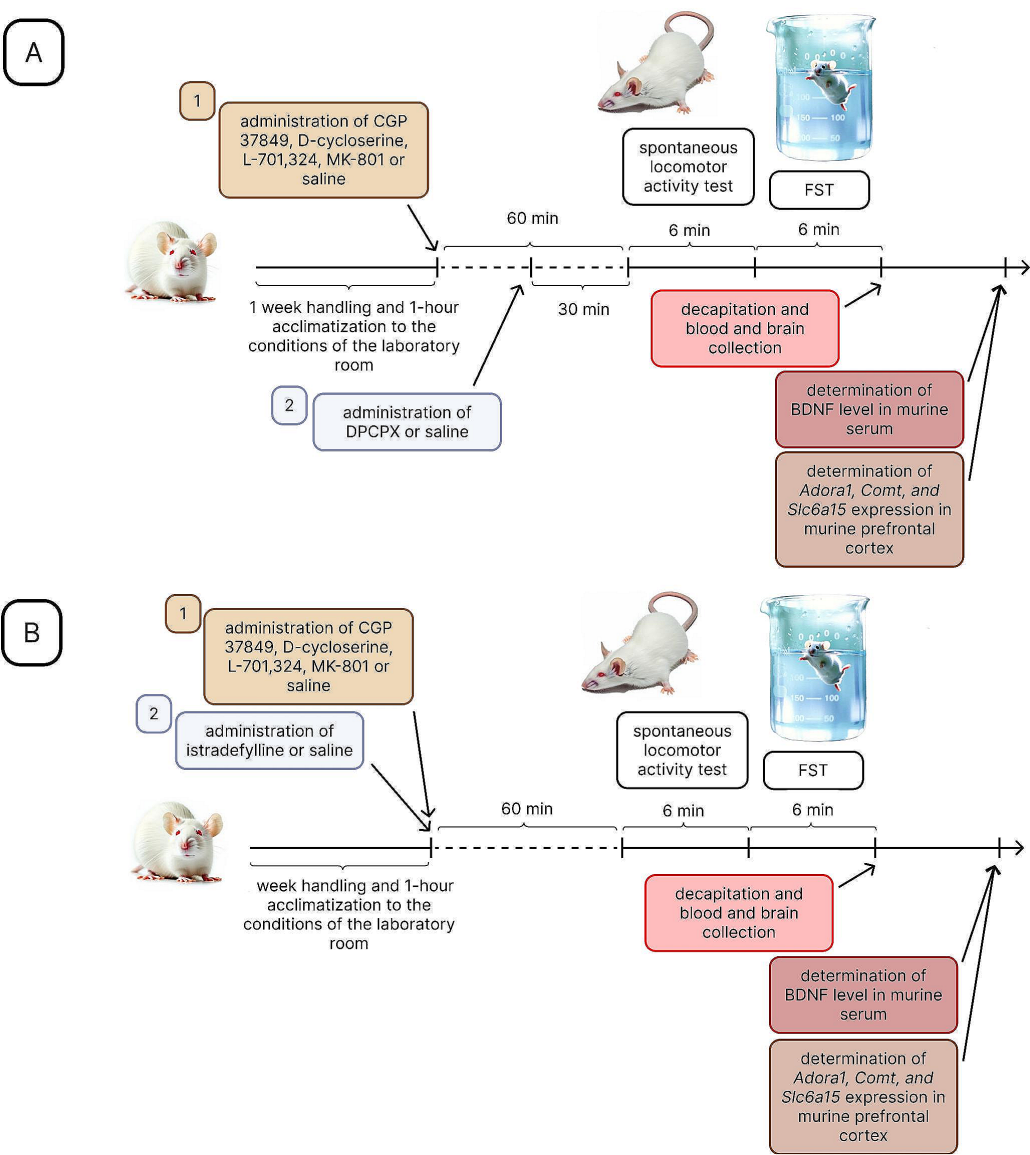
Research schedule. (**A**) Experiments with DPCPX; (**B**) Experiments with istradefylline. *Abbreviations Adora1*, adenosine A1 receptor gene; BDNF, brain–derived neurotrophic factor; *Comt*, catechol–*O*–methyl–transferase gene; FST, forced swim test; *Slc6a15*, solute carrier family 6 (neurotransmitter transporter) member 15 gene

The treatment schedule (Scheme 1) and applied doses of the tested substances were selected based on previous studies conducted by our team and preliminary research carried out in our laboratory, as referenced in the following publications [32, 35–37].

#### Forced swim test (FST)

The FST was conducted by the previously described methodology [32]. Each animal was placed in a glass cylinder filled with water at a temperature of 23–25 °C for 6 min. Following a 2-min period of acclimatization, the duration of immobility was recorded for the subsequent 4 min of the test. The animal was considered immobile when it exhibited only those movements that were necessary to maintain its head above the water.

The experiment was recorded by tablets (Samsung Galaxy Table 4, Samsung Electronics, Seoul, South Korea) and then the immobility time was measured manually by two blinded researchers. The FST results were presented as the arithmetic mean of the immobility time (in seconds) of the mice, with the standard error of the mean (SEM) for each experimental group (*n* = 10 mice).

#### Spontaneous locomotor activity test

The spontaneous locomotor activity test was conducted with the previously described methodology [32]. Opto-Varimex-4 Auto-Track (Columbus Instruments, Columbus, OH, USA) was used to record the motility of each mouse. The spontaneous locomotor activity was recorded of over 4 min to before the commencement of the FST.

The spontaneous locomotor activity test results were shown as the arithmetic average distance traveled (cm) by mice for 4 min of test ± SEM for each experimental group (*n* = 8–10 mice).

#### Determination of BDNF level in murine serum

Following the completion of the behavioral tests, the mice (*n* = 6 mice per group) were humanely euthanized by decapitation. The blood samples were collected into polyethylene tubes and left at room temperature until further processing. The clotted blood was then centrifuged for 10 min at 5,000 × g. The serum was subsequently transferred into Eppendorf tubes and placed in a freezer at a temperature of − 25 °C, where it was stored until the BDNF assays were performed. BNDF levels in murine serum were evaluated using an ELISA diagnostic kit following the manufacturer’s leaflet.

#### Determination of gene expression in murine prefrontal cortex

Directly after decapitation of animals (*n* = 6 mice per group), their brains were removed and placed in chilled saline (2–8 °C). Then, the prefrontal cortex was dissected out, put into polyethylene tubes, and placed in a freezer (− 80 °C), where it was stored until molecular analyses were performed.

TRIzol reagent was employed for the purpose of isolating total RNA from the prefrontal cortex of mice. The procedure was conducted in accordance with the instructions provided in the manufacturer’s leaflet. The prefrontal cortex was initially homogenised with TRIzol reagent (500 µl) at a concentration of 30 mg. Then 100 µl of chloroform was added to each probe. The resulting mixture was then incubated for a period of three minutes and subsequently subjected to centrifugation (4 °C, 12,000 × g, 15 min). The aqueous phase containing the RNA was transferred to a clean tube, to which 250 µl of isopropanol was added. The mixture was then incubated for 10 min and centrifuged (4 °C, 12,000 × g, 15 min). The resulting white pellet at the bottom of the tube was washed with 500 µl of 75% ethanol, dried, and subsequently dissolved in 50 µl of RNase-free water. The purity and concentration of the RNA were determined using a NanoDrop Maestro Nano spectrophotometer (Maestrogen, Hsinchu, Taiwan). For subsequent analysis, the high-purity RNA exhibited an A260/280 ratio within the range of 1.8 to 2.0.

The cDNA synthesis was conducted in accordance with the instructions provided in the manufacturer’s leaflet for a high-capacity cDNA reverse transcription kit. Firstly, a reaction mixture was prepared containing 10 µl of isolated RNA (200 ng/µl), 2 µl of 10X RT Buffer, 0.8 µl of 25X dNTP Mix (100 mM), 2 µl of 10X RT random primers, 1 µl of MultiScribe reverse transcriptase (50 U/µl), 0.5 µl of RNAse inhibitor (40 U/µl) and 3.2 µl of RNAse-free water, in triplicate. The received mixture was subjected to the following reaction conditions: (1) 10 min at 25 °C, (2) 120 min at 37 °C, and (3) 5 min at 85 °C to finish the process. Obtained cDNA was placed in a freezer (− 20 °C), where it was stored until further analyzes were performed.

The real-time PCR reaction, ΔΔCt method, and *Hprt* and *Tbp* as endogenous controls were used to measure the relative expression of *Adora1*, *Comt*, and *Slc6a15*. The reference genes were selected based on preliminary analysis, which aimed at determining variability under experimental conditions, and according to the requirements of MIQE Guidelines [38]. Firstly, a reaction mixture containing 1 µl of the cDNA (5 ng), 10 µl of Fast Probe qPCR Master Mix (2×), 9 µl of RNase–free water, 0.5 µl of ROX Solution (50 nM), and 0.5 µM of gene–specific TaqMan probe (Table 1) was prepared. The real-time PCR reaction was carried out in triplicates using the 7500 Fast Real–Time PCR System (Applied Biosystems, Foster City, CA, USA) and the following conditions: 1 cycle: 3 min in 95 °C and 40 cycles: 10 s in 95 °C and 30 s in 60 °C. To remove any outliers, before performing ΔΔCt calculations and determining the fold change of mRNA levels of selected genes the data quality control based on amplification T_m_ and C_t_ values were performed. The results were shown as the RQ value (RQ = 2^−ΔΔCt^).

**Table 1:** Names and symbols of gene names, GenBank reference sequence accession numbers, assay IDs, and amplicon lengths

Gene name	Gene symbol	GenBank ref. seq.	Assay ID	Amplicon Length
Adenosine A1 receptor	*Adora1*	NM_001008533.3 NM_001039510.2 NM_001282945.1	Mm01308023_m1	58
Catechol–*O*–methyl– transferase	*Comt*	NM_001111062.1 NM_001111063.1 NM_007744.3	Mm00514377_m1	97
Hypoxanthine guanine phosphoribosyl transferase	*Hprt*	NM_013556.2	Mm00446968_m1	65
Solute carrier family 6 (neurotransmitter transporter), member 15	*Slc6a15*	NM_001252330.1 NM_175328.3	Mm00558415_m1	84
TATA box binding protein	*Tbp*	NM_013684.3	Mm00446974_m1	105

### Statistical analysis

Before performing the appropriate statistical analysis all data were subjected to a preliminary screening process: (1) outliers were identified using the Grubbs’s test (https://www.graphpad.com/quickcalcs/Grubbs1.cfm)– the outliers were excluded from the two-way ANOVA analysis, and (2) the assumption of parametric tests– the obtained results met the assumptions of parametric tests, including homoscedasticity and normal distribution.

All results were subjected to two-way ANOVA with Bonferroni’s post hoc test (Prism 6, GraphPad, San Diego, CA, USA). Two-way ANOVA considered the following independent variables: (1) treatment with selective adenosine A1 or A2A receptor antagonist, and (2) treatment with NMDA receptor ligand. Additionally, analysis of variance also allowed for the identification of interactions between the tested agents when administered concomitantly. Significance was determined by a *p*-value of less than 0.05.

## Results

### Effects of a combined administration of selective adenosine receptor antagonists and NMDA receptor ligands on mice behavior in the FST

*DPCPX and L–701*,*324 combination*. Two-way ANOVA indicated a significant effect of DPCPX (F_1,36_=5.32, *p* = 0.0269) and L–701,324 (F_1,36_=18.79, *p* = 0.0001), and a significant DPCPX × L–701,324 interaction (F_1,36_=13.67, *p* = 0.0007) on immobility time in the FST.

**Fig. 1:**
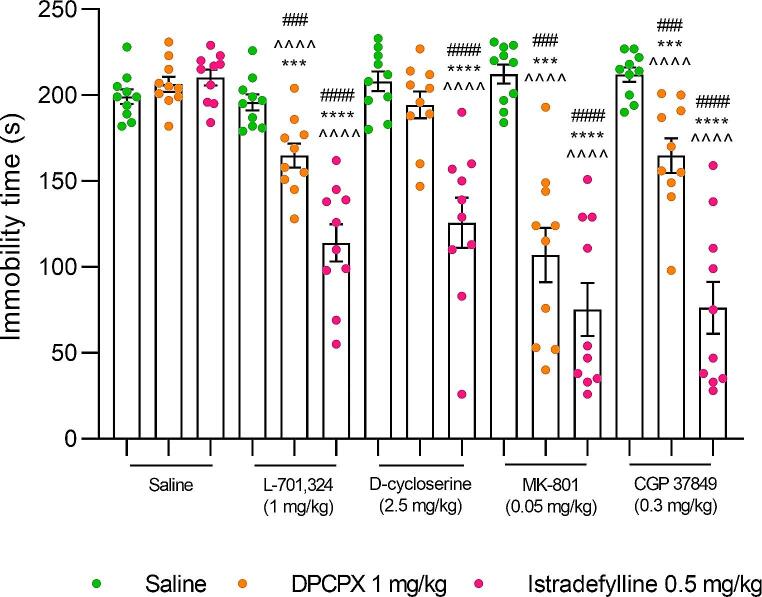
An influence of concomitant administration of (**A**) a selective A1 receptor antagonist– DPCPX, and (**B**) selective A2A receptor antagonist– istradefylline, with NMDA receptor ligands on mice behaviour in the forced swim test. Animals received *intraperitoneally* (*ip*) L–701,324 (1 mg/kg), D–cycloserine (2.5 mg/kg), CGP 37849 (0.3 mg/kg) or MK–801 (0.05 mg/kg) 60 min before the behavioural experiment. Istradefylline (0.5 mg/kg) was given orally (*po*) 60 min before the test, whereas DPCPX (1 mg/kg) was administered *ip* 30 min before the test. Mice from the control group received two administrations of saline, i.e. the first one *po* 60 min and the second *ip* 30 min prior behavioral testing. The following drug combinations were used: (1) saline + saline, (2) saline + DPCPX, (3) saline + istradefylline, (4) L–701,324 + saline, (5) L–701,324 + DPCPX, (6) L–701,324 + istradefylline, (7) D–cycloserine + saline, (8) D–cycloserine + DPCPX, (9) D–cycloserine + istradefylline, (10) CGP 37849 + saline, (11) CGP 37849 + DPCPX, (12) CGP 37849 + istradefylline, (13) MK–801 + saline, (14) MK–801 + DPCPX, (15) MK–801 + istradefylline. The values represent mean (s) ± SEM (*n* = 10 mice per group). Significance: ***p < 0.001, ****p < 0.0001 versus saline + saline-treated group; ^^^^*p* < 0.0001 versus saline + DPCPX- or saline + istradefylline-treated group (respectively), ^###^*p* < 0.001, ^####^*p* < 0.0001 versus saline + respective NMDA receptor ligand-treated group. Data were analysed by two-way ANOVA with Bonferroni’s post hoc test.

A combined administration of DPCPX and L–701,324 resulted in a statistically significant reduction in the duration of mice immobility compared to the saline- (*p* < 0.001), DPCPX- (*p* < 0.0001), and L–701,324-treated group (*p* < 0.001) (Fig. 1).

*DPCPX and D–cycloserine combination.* Two-way ANOVA indicated no significant effect of DPCPX (F_1,36_=0.04, *p* = 0.8452) and D–cycloserine (F_1,36_=0.01, *p* = 0.9351), and a significant DPCPX × D–cycloserine interaction (F_1,36_=4.27, *p* = 0.0461) on immobility time in the FST.

A combined administration of DPCPX and D–cycloserine did not result in a statistically significant reduction in the duration of mice immobility compared to the saline- (*p* > 0.05), DPCPX- (*p* > 0.05), and D–cycloserine-treated group (*p* > 0.05) (Fig. 1).

*DPCPX and MK–801 combination.* Two-way ANOVA indicated a significant effect of DPCPX (F_1,36_=26.18, *p* < 0.0001) and MK–801 (F_1,36_=20.10, *p* < 0.0001), and a significant DPCPX × MK–801 interaction (F_1,36_=40.45, *p* < 0.0001) on immobility time in the FST.

A combined administration of DPCPX and MK–801 resulted in a statistically significant reduction in the duration of mice immobility compared to the saline- (*p* < 0.001), DPCPX- (*p* < 0.0001), and MK–801-treated group (*p* < 0.001) (Fig. 1).

*DPCPX and CGP 37849 combination.* Two-way ANOVA indicated a significant effect of DPCPX (F_1,36_=7.31, *p* = 0.0104) and no effect of CGP 37849 (F_1,36_=3.50, *p* = 0.0695), and a significant DPCPX × CGP 37849 interaction (F_1,36_=19.63, *p* < 0.0001) on immobility time in the FST.

A combined administration of DPCPX and CGP 37849 resulted in a statistically significant reduction in the duration of mice immobility compared to the saline- (*p* < 0.001), DPCPX- (*p* < 0.0001), and CGP 37849-treated group (*p* < 0.001) (Fig. 1).

*Istradefylline and L–701*,*324 combination*. Two-way ANOVA indicated a significant effect of istradefylline (F_1,36_=27.79, *p* < 0.0001) and L–701,324 (F_1,36_=55.39, *p* < 0.0001), and a significant istradefylline × L–701,324 interaction (F_1,36_=48.29, *p* < 0.0001) on immobility time in the FST.

A combined administration of istradefylline and L–701,324 resulted in a statistically significant reduction in the duration of mice immobility compared to the saline- (*p* < 0.0001), istradefylline- (*p* < 0.0001), and L–701,324-treated group (*p* < 0.0001) (Fig. 1).

*Istradefylline and D–cycloserine combination.* Two-way ANOVA indicated a significant effect of istradefylline (F_1,36_=14.84, *p* = 0.0005) and D–cycloserine (F_1,36_=16.85, *p* = 0.0002), and a significant istradefylline × D–cycloserine interaction (F_1,36_=31.47, *p* < 0.0001) on immobility time in the FST.

A combined administration of istradefylline and D–cycloserine resulted in a statistically significant reduction in the duration of mice immobility compared to the saline- (*p* < 0.0001), istradefylline- (*p* < 0.0001), and D–cycloserine-treated group (*p* < 0.0001) (Fig. 1).

*Istradefylline and MK–801 combination.* Two-way ANOVA indicated a significant effect of istradefylline (F_1,36_=45.74, *p* < 0.0001) and MK–801 (F_1,36_=42.92, *p* < 0.0001), and a significant istradefylline × MK–801 interaction (F_1,36_=71.65, *p* < 0.0001) on immobility time in the FST.

A combined administration of istradefylline and MK–801 resulted in a statistically significant reduction in the duration of mice immobility compared to the saline- (*p* < 0.0001), istradefylline- (*p* < 0.0001), and MK–801-treated group (*p* < 0.0001) (Fig. 1).

*Istradefylline and CGP 37849 combination.* Two-way ANOVA indicated a significant effect of istradefylline (F_1,36_=47.82, *p* < 0.0001) and CGP 37849 (F_1,36_=45.31, *p* < 0.0001), and a significant istradefylline × CGP 37849 interaction (F_1,36_=75.24, *p* < 0.0001) on immobility time in the FST.

A combined administration of istradefylline and CGP 37849 resulted in a statistically significant reduction in the duration of mice immobility compared to the saline- (*p* < 0.0001), istradefylline- (*p* < 0.0001), and CGP 37849-treated group (*p* < 0.0001) (Fig. 1).

### Effects of a combined administration of selective adenosine receptor antagonists and NMDA receptor ligands on the spontaneous locomotor activity of mice

*DPCPX and L–701*,*324 combination*. Two-way ANOVA indicated no significant effect of DPCPX (F_1,36_=0.63, *p* = 0.4309) and L–701,324 (F_1,36_=3.15, *p* = 0.0843), and no significant DPCPX × L–701,324 interaction (F_1,36_=1.11, *p* = 0.2985) on the spontaneous locomotor activity of mice (Table 2).

**Table 2:** An influence of concomitant administration of a selective A1 receptor antagonist– DPCPX, and a selective A2A receptor antagonist– istradefylline, with NMDA receptor ligands on the motility of mice for 4 min of the spontaneous locomotor activity test

Treatment (mg/kg)	Spontaneous locomotor activity (cm)	Treatment (mg/kg)	Spontaneous locomotor activity (cm)
Saline + Saline	1303.7 ± 64.7	L–701,324 (1) + DPCPX (1)	1120.5 ± 53.8
Saline + DPCPX (1)	1322.1 ± 85.7	L–701,324 (1) + Istradefylline (0.5)	1452.0 ± 138
Saline + Istradefylline (0.5)	1303.9 ± 85.8	D–cycloserine (2.5) + DPCPX (1)	1128.9 ± 67.7
Saline + L–701,324 (1)	1252.4 ± 76.7	D–cycloserine (2.5) + Istradefylline (0.5)	1282.6 ± 126
Saline + D–cycloserine (2.5)	1181.7 ± 56.0	MK–801 (0.05) + DPCPX (1)	1351.5 ± 42.2
Saline + MK–801 (0.05)	1347.9 ± 80.2	MK–801 (0.05) + Istradefylline (0.5)	1445.8 ± 205
Saline + CGP 37849 (0.3)	1115.6 ± 72.3	CGP 37849 (0.3) + DPCPX (1)	1029.3 ± 80.4
		CGP 37849 (0.3) + Istradefylline (0.5)	1229.6 ± 102

Animals received *intraperitoneally* (*ip*) L–701,324 (1 mg/kg), D–cycloserine (2.5 mg/kg), CGP 37849 (0.3 mg/kg) or MK–801 (0.05 mg/kg) 60 min before the behavioural experiment. Istradefylline (0.5 mg/kg) was given orally (*po*) 60 min before the test, whereas DPCPX (1 mg/kg) was administered *ip* 30 min before the test. Mice from the control group received two administrations of saline, i.e. the first one *po* 60 min and the second *ip* 30 min prior behavioral testing. The spontaneous locomotor activity was measured for 4 min before the FST. The values represent mean (cm) ± SEM. Significance: *p* > 0.05. Data were analyzed by two-way ANOVA with Bonferroni’s post hoc test.

*DPCPX and D–cycloserine combination.* Two-way ANOVA indicated no significant effect of DPCPX (F_1,34_=0.22, *p* = 0.6410) and D–cycloserine (F_1,34_=2.74, *p* = 0.1073), and no significant DPCPX × D–cycloserine interaction (F_1,34_=1.61, *p* = 0.2128) on the spontaneous locomotor activity of mice (Table 2).

*DPCPX and MK–801 combination.* Two-way ANOVA indicated no significant effect of DPCPX (F_1,35_=0.77, *p* = 0.3875) and MK–801 (F_1,35_=1.58, *p* = 0.2175), and no significant DPCPX × MK–801 interaction (F_1,35_=0.67, *p* = 0.4171) on the spontaneous locomotor activity of mice (Table 2).

*DPCPX and CGP 37849 combination.* Two-way ANOVA indicated no significant effect of DPCPX (F_1,35_=0.04, *p* = 0.8442) and a significant effect of CGP 37849 (F_1,35_=7.01, *p* = 0.0121), and no significant DPCPX × CGP 37849 interaction (F_1,35_=1.92, *p* = 0.1743) on the spontaneous locomotor activity of mice (Table 2).

*Istradefylline and L–701*,*324 combination*. Two-way ANOVA indicated no significant effect of istradefylline (F_1,36_=1.09, *p* = 0.3028) and L–701,324 (F_1,36_=0.26, *p* = 0.6156), and no significant istradefylline × L–701,324 interaction (F_1,36_=1.09, *p* = 0.3037) on the spontaneous locomotor activity of mice (Table 2).

*Istradefylline and D–cycloserine combination.* Two-way ANOVA indicated no significant effect of istradefylline (F_1,36_=1.36, *p* = 0.2515) and D–cycloserine (F_1,36_=0.08, *p* = 0.7847), and no significant istradefylline × D–cycloserine interaction (F_1,36_=5.84, *p* = 0.9809) on the spontaneous locomotor activity of mice (Table 2).

*Istradefylline and MK–801 combination.* Two-way ANOVA indicated no significant effect of istradefylline (F_1,36_=1.50, *p* = 0.2290) and MK–801 (F_1,36_=2.53, *p* = 0.1208), and no significant istradefylline × MK–801 interaction (F_1,36_=0.18, *p* = 0.6771) on the spontaneous locomotor activity of mice (Table 2).

*Istradefylline and CGP 37849 combination.* Two-way ANOVA indicated no significant effect of istradefylline (F_1,36_=1.76, *p* = 0.1926) and CGP 37849 (F_1,36_=1.09, *p* = 0.3033), and no significant istradefylline × CGP 37849 interaction (F_1,36_=0.01, *p* = 0.9143) on the spontaneous locomotor activity of mice (Table 2).

3.3. Effects of a combined administration of selective adenosine receptor antagonists and NMDA receptor ligands on the BDNF level in mice serum.

**Fig. 2:**
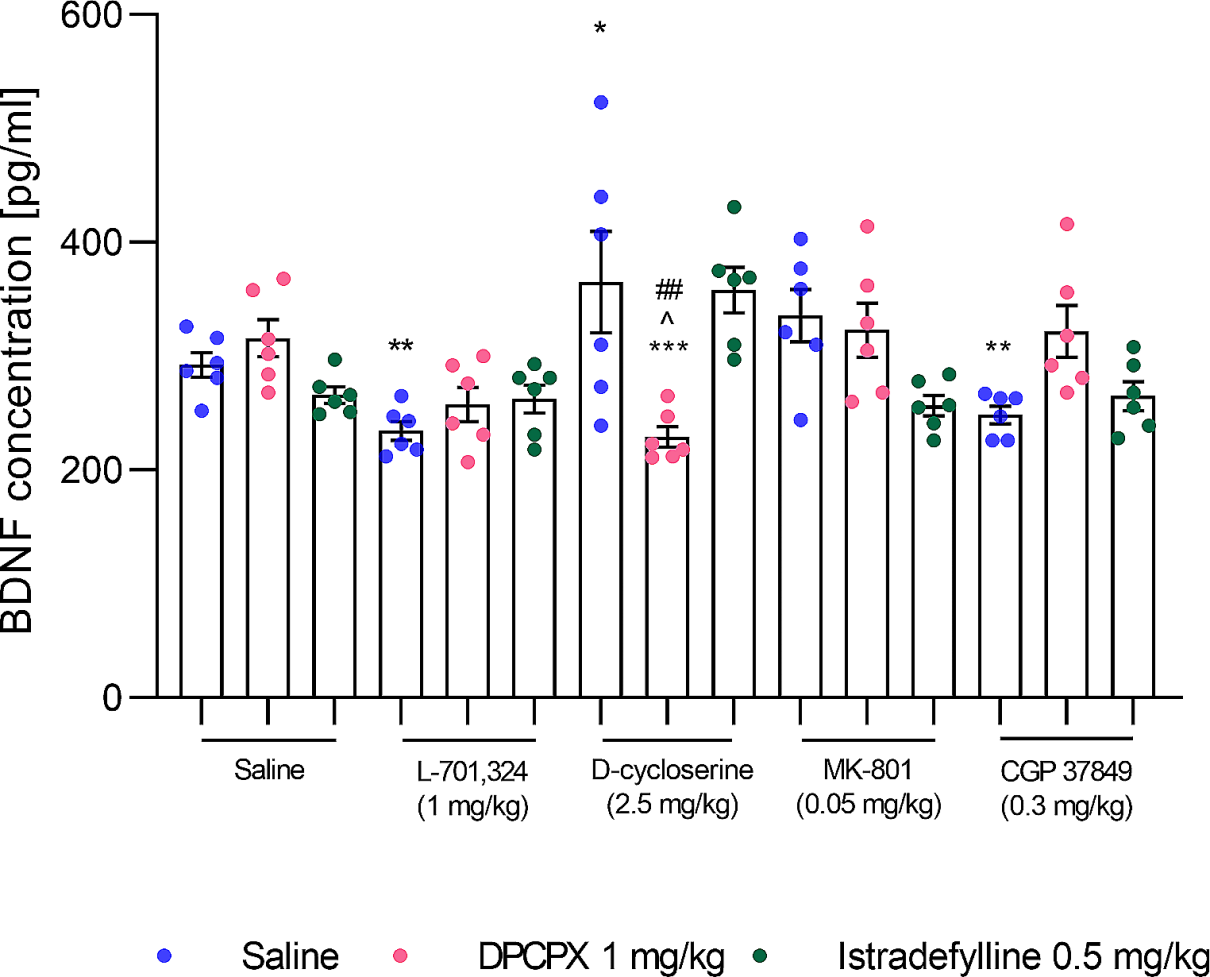
An influence of concomitant administration of a selective A1 receptor antagonist– DPCPX, and selective A2A receptor antagonist– istradefylline, with NMDA receptor ligands on the BDNF levels in the murine serum. Animals received *intraperitoneally* (*ip*) L–701,324 (1 mg/kg), D–cycloserine (2.5 mg/kg), CGP 37849 (0.3 mg/kg) or MK–801 (0.05 mg/kg) 60 min before decapitation. Istradefylline (0.5 mg/kg) was given orally (*po*) 60 min before the procedure, whereas DPCPX (1 mg/kg) was administered *ip* 30 min before the procedure. Mice from the control group received two administrations of saline, i.e. the first one *po* 60 min and the second *ip* 30 min prior decapitation.The following drug combinations were used: (1) saline + saline, (2) saline + DPCPX, (3) saline + istradefylline, (4) L–701,324 + saline, (5) L–701,324 + DPCPX, (6) L–701,324 + istradefylline, (7) D–cycloserine + saline, (8) D–cycloserine + DPCPX, (9) D–cycloserine + istradefylline, (10) CGP 37849 + saline, (11) CGP 37849 + DPCPX, (12) CGP 37849 + istradefylline, (13) MK–801 + saline, (14) MK–801 + DPCPX, (15) MK–801 + istradefylline. The values represent mean (pg/ml) ± SEM (*n* = 6 mice per group). Significance: **p < 0.01, ***p < 0.001 versus saline + saline-treated group; ^*p* < 0.05 versus saline + DPCPX-treated group; ^##^*p* < 0.01 versus saline + respective NMDA receptor ligand-treated group. Data were analysed by two-way ANOVA with Bonferroni’s post hoc test.

*DPCPX and L–701*,*324 combination.* Two-way ANOVA indicated no significant effect of DPCPX (F_1,20_=3.16, *p* = 0.0908) and a significant effect of L–701,324 (F_1,20_=19.78, *p* = 0.0002), and no significant DPCPX × L–701,324 interaction (F_1,20_=4.75, *p* > 0.9999) on the BDNF level in mice serum (Fig. 2).

*DPCPX and D–cycloserine combination.* Two-way ANOVA indicated a significant effect of DPCPX (F_1,20_=5.19, *p* = 0.0338) and D–cycloserine (F_1,20_=16.85, *p* = 0.0002), and a significant DPCPX × D–cycloserine interaction (F_1,20_=10.33, *p* = 0.0044) on the BDNF level in mice serum.

A combined administration of DPCPX and D–cycloserine significantly decreased the BDNF level in mice serum compared to the saline- (*p* < 0.001), DPCPX- (*p* < 0.05), and D–cycloserine-treated group (*p* < 0.01) (Fig. 2). Additionally, a single administration of D–cycloserine significantly increased the level of BDNF in mice serum when compared to the saline-treated group (*p* < 0.05).

*DPCPX and MK–801 combination.* Two-way ANOVA indicated no significant effect of DPCPX (F_1,20_=0.07, *p* = 0.7884) and MK–801 (F_1,20_=1.69, *p* = 0.2085), and no significant DPCPX × MK–801 interaction (F_1,20_=0.86, *p* = 0.3643) on the BDNF level in mice serum (Fig. 2).

*DPCPX and CGP 37849 combination.* Two-way ANOVA indicated a significant effect of DPCPX (F_1,20_=9.68, *p* = 0.0055) and no significant effect of CGP 37849 (F_1,20_=1.51, *p* = 0.2339), and no significant DPCPX × CGP 37849 interaction (F_1,20_=2.61, *p* = 0.1220) on the BDNF level in mice serum (Fig. 2).

*Istradefylline and L–701*,*324 combination.* Two-way ANOVA indicated no significant effect of istradefylline (F_1,20_=3.47, *p* = 0.9536) and a significant effect of L–701,324 (F_1,20_=9.64, *p* = 0.0056), and a significant istradefylline × L–701,324 interaction (F_1,20_=7.57, *p* = 0.0123) on the BDNF level in mice serum.

A combined administration of istradefylline and L–701,324 had no statistically significant effect on the BDNF level in mice serum compared to the saline-, istradefylline-, and L–701,324-treated group (*p* > 0.05) (Fig. 2). Additionally, a single administration of L–701,324 significantly decreased the level of BDNF in mice serum when compared to the saline-treated group (*p* < 0.01), and istradefylline attenuated this effect.

*Istradefylline and D–cycloserine combination.* Two-way ANOVA indicated no significant effect of istradefylline (F_1,20_=0.45, *p* = 0.5108) and a significant effect of D–cycloserine (F_1,20_=10.64, *p* = 0.0039), and no significant istradefylline × D–cycloserine interaction (F_1,20_=0.15, *p* = 0.7036) on the BDNF level in mice serum (Fig. 2).

*Istradefylline and MK–801 combination.* Two-way ANOVA indicated a significant effect of istradefylline (F_1,20_=14.21, *p* = 0.0012) and no significant effect of MK–801 (F_1,20_=1.46, *p* = 0.2408), and no significant istradefylline × MK–801 interaction (F_1,20_=3.48, *p* = 0.0770) on the BDNF level in mice serum (Fig. 2).

*Istradefylline and CGP 37849 combination.* Two-way ANOVA indicated a significant effect of istradefylline (F_1,20_=5.26, *p* = 0.0328) and no significant effect of CGP 37849 (F_1,20_=0.28, *p* = 0.6042), and a significant istradefylline × CGP 37849 interaction (F_1,20_=4.80, *p* = 0.0404) on the BDNF level in mice serum.

A combined administration of istradefylline and CGP 37849 had no statistically significant effect on the BDNF level in mice serum compared to the saline-, istradefylline-, and CGP 37849-treated group (*p* > 0.05) (Fig. 2). Additionally, a single administration of CGP 37849 significantly decreased the level of BDNF in mice serum when compared to the saline-treated group (*p* < 0.01), and istradefylline blocked this effect.

### Effects of a combined administration of selective adenosine receptor antagonists and NMDA receptor ligands on gene expression in murine prefrontal cortex

#### Adora1 gene expression

*DPCPX and L–701*,*324 combination.* Two-way ANOVA indicated a significant effect of DPCPX (F_1,20_=184.6, *p* < 0.0001) and L–701,324 (F_1,20_=12.68, *p* = 0.0020), and a significant DPCPX × L–701,324 interaction (F_1,20_=384.0, *p* < 0.0001) on *Adora1* expression in murine prefrontal cortex.

**Fig. 3:**
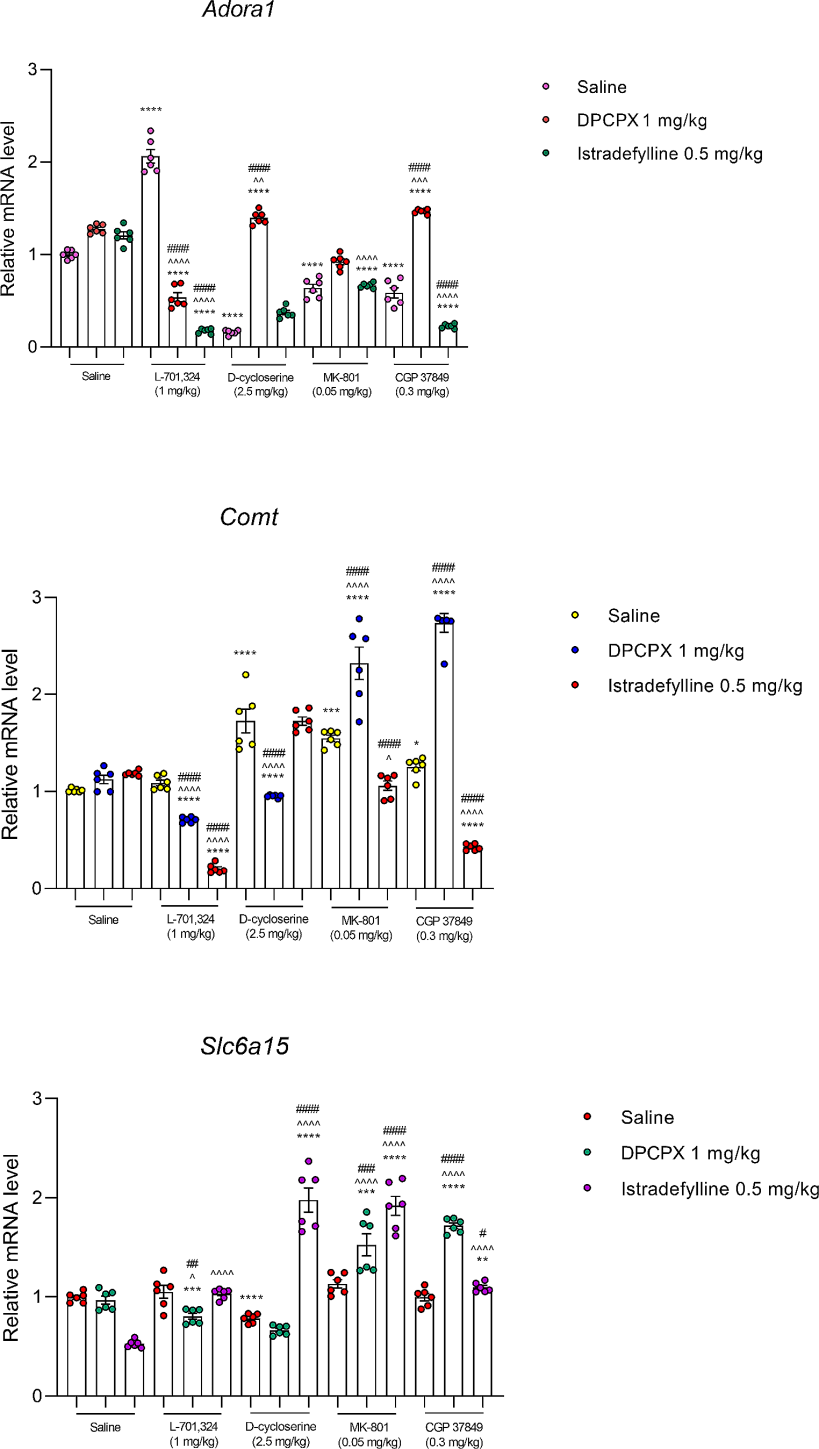
An influence of concomitant administration of DPCPX and istradefylline with NMDA receptor ligands on the *Adora1*,* Comt* and *Slc6a15* relative mRNA level in murine prefrontal cortex. Animals received *intraperitoneally* (*ip*) L–701,324 (1 mg/kg), D–cycloserine (2.5 mg/kg), CGP 37849 (0.3 mg/kg) or MK–801 (0.05 mg/kg) 60 min before decapitation. Istradefylline (0.5 mg/kg) was given orally (*po*) 60 min before the procedure, whereas DPCPX (1 mg/kg) was administered *ip* 30 min before the procedure. Mice from the control group received two administrations of saline, i.e. the first one *po* 60 min and the second *ip* 30 min prior decapitation.The following drug combinations were used: (1) saline + saline, (2) saline + DPCPX, (3) saline + istradefylline, (4) L–701,324 + saline, (5) L–701,324 + DPCPX, (6) L–701,324 + istradefylline, (7) D–cycloserine + saline, (8) D–cycloserine + DPCPX, (9) D–cycloserine + istradefylline, (10) CGP 37849 + saline, (11) CGP 37849 + DPCPX, (12) CGP 37849 + istradefylline, (13) MK–801 + saline, (14) MK–801 + DPCPX, (15) MK–801 + istradefylline. The values represent mean (relative mRNA level) ± SEM (*n* = 6 mice per group). Significance: *p < 0.05, **p < 0.01, ***p < 0.001, ****p < 0.0001 versus saline + saline-treated group; ^*p* < 0.05, ^^^*p* < 0.001, ^^^^*p* < 0.0001 versus saline + DPCPX- or saline + istradefylline-treated group (respectively); ^#^*p* < 0.05, ^##^*p* < 0.01, ^###^*p* < 0.001, ^####^*p* < 0.0001 versus saline + respective NMDA receptor ligand-treated group. Data were analysed by two-way ANOVA with Bonferroni’s post hoc test.

A combined administration of DPCPX and L–701,324 significantly decreased *Adora1* expression in murine prefrontal cortex compared to the saline- (*p* < 0.0001), DPCPX- (*p* < 0.0001), and L–701,324-treated group (*p* < 0.0001) (Fig. 3). Additionally, a single administration of L–701,324 significantly increased *Adora1* expression in murine prefrontal cortex when compared to the saline-treated group (*p* < 0.0001), and DPCPX blocked this effect and even reduced *Adora1* expression below the value reported for the control group.

*DPCPX and D–cycloserine combination.* Two-way ANOVA indicated a significant effect of DPCPX (F_1,20_=1380, *p* < 0.0001) and D–cycloserine (F_1,20_=309.7, *p* < 0.0001), and a significant DPCPX × D–cycloserine interaction (F_1,20_=558.5, *p* < 0.0001) on *Adora1* expression in murine prefrontal cortex.

A combined administration of DPCPX and D–cycloserine significantly increased *Adora1* expression in murine prefrontal cortex compared to the saline- (*p* < 0.0001), DPCPX- (*p* < 0.01), and D–cycloserine-treated group (*p* < 0.0001) (Fig. 3). Additionally, a single administration of D–cycloserine significantly decreased *Adora1* expression in murine prefrontal cortex when compared to the saline-treated group (*p* < 0.0001), and DPCPX blocked this effect and even increased *Adora1* expression above the value reported for the control group.

*DPCPX and MK–801 combination.* Two-way ANOVA indicated a significant effect of DPCPX (F_1,20_=91.81, *p* < 0.0001) and MK–801 (F_1,20_=148.8, *p* < 0.0001), and no significant DPCPX × MK–801 interaction (F_1,20_=0.03006, *p* = 0.8641) on *Adora1* expression in murine prefrontal cortex (Fig. 3).

*DPCPX and CGP 37849 combination.* Two-way ANOVA indicated a significant effect of DPCPX (F_1,20_=350.8, *p* < 0.0001) and CGP 37849 (F_1,20_=13.08, *p* = 0.0017), and a significant DPCPX × CGP 37849 interaction (F_1,20_=95.92, *p* < 0.0001) on *Adora1* expression in murine prefrontal cortex.

A combined administration of DPCPX and CGP 37849 significantly increased *Adora1* expression in murine prefrontal cortex compared to the saline- (*p* < 0.0001), DPCPX- (*p* < 0.001), and CGP 37849-treated group (*p* < 0.0001) (Fig. 3). Additionally, a single administration of CGP 37849 significantly decreased *Adora1* expression in murine prefrontal cortex when compared to the saline-treated group (*p* < 0.0001), and DPCPX blocked this effect and even increased *Adora1* expression above the value reported for the control group.

*Istradefylline and L–701*,*324 combination.* Two-way ANOVA indicated a significant effect of istradefylline (F_1,20_=382.8, *p* < 0.0001) and no effect of L–701,324 (F_1,20_=0.1287, *p* = 0.7235), and a significant istradefylline × L–701,324 interaction (F_1,20_=594.4, *p* < 0.0001) on *Adora1* expression in murine prefrontal cortex.

A combined administration of istradefylline and L–701,324 significantly decreased *Adora1* expression in murine prefrontal cortex compared to the saline- (*p* < 0.0001), istradefylline- (*p* < 0.0001), and L–701,324-treated group (*p* < 0.0001) (Fig. 3). Additionally, a single administration of L–701,324 significantly increased *Adora1* expression in murine prefrontal cortex when compared to the saline-treated group (*p* < 0.0001), and istradefylline blocked this effect and even reduced *Adora1* expression below the value reported for the control group.

*Istradefylline and D–cycloserine combination.* Two-way ANOVA indicated a significant effect of istradefylline (F_1,20_=71.65, *p* < 0.0001) and D–cycloserine (F_1,20_=1130, *p* < 0.0001), and no significant istradefylline × D–cycloserine interaction (F_1,20_=0.02471, *p* = 0.8767) on *Adora1* expression in murine prefrontal cortex (Fig. 3).

*Istradefylline and MK–801 combination.* Two-way ANOVA indicated a significant effect of istradefylline (F_1,20_=14.98, *p* = 0.0010) and MK–801 (F_1,20_=219.1, *p* < 0.0001), and a significant istradefylline × MK–801 interaction (F_1,20_=8.494, *p* = 0.0086) on *Adora1* expression in murine prefrontal cortex (Fig. 3).

A combined administration of istradefylline and MK–801 significantly decreased *Adora1* expression in murine prefrontal cortex compared to the saline- (*p* < 0.0001), and istradefylline-treated group (*p* < 0.0001) (Fig. 3). Additionally, a single administration of MK–801 significantly decreased *Adora1* expression in murine prefrontal cortex when compared to the saline-treated group (*p* < 0.0001) and istradefylline had no impact on this effect.

*Istradefylline and CGP 37849 combination.* Two-way ANOVA indicated a significant effect of istradefylline (F_1,20_=4.795, *p* = 0.0406) and a significant effect of CGP 37849 (F_1,20_=395.5, *p* < 0.0001), and a significant istradefylline × CGP 37849 interaction (F_1,20_=65.43, *p* < 0.0001) on *Adora1* expression in murine prefrontal cortex.

A combined administration of istradefylline and CGP 37849 significantly decreased *Adora1* expression in murine prefrontal cortex compared to the saline- (*p* < 0.0001), istradefylline- (*p* < 0.0001), and CGP 37849-treated group (*p* < 0.0001) (Fig. 3). Additionally, a single administration of CGP 37849 significantly decreased *Adora1* expression in murine prefrontal cortex when compared to the saline-treated group (*p* < 0.0001), and istradefylline enhanced this effect.

#### Comt gene expression

*DPCPX and L–701*,*324 combination.* Two-way ANOVA indicated a significant effect of DPCPX (F_1,20_=23.91, *p* < 0.0001) and L–701,324 (F_1,20_=38.34, *p* < 0.0001), and a significant DPCPX × L–701,324 interaction (F_1,20_=80.74, *p* < 0.0001) on *Comt* expression in murine prefrontal cortex.

A combined administration of DPCPX and L–701,324 significantly decreased *Comt* expression in murine prefrontal cortex compared to the saline- (*p* < 0.0001), DPCPX- (*p* < 0.0001), and L–701,324-treated group (*p* < 0.0001) (Fig. 3).

*DPCPX and D–cycloserine combination.* Two-way ANOVA indicated a significant effect of DPCPX (F_1,20_=25.88, *p* < 0.0001) and D–cycloserine (F_1,20_=17.22, *p* = 0.0005), and a significant DPCPX × D–cycloserine interaction (F_1,20_=46.53, *p* < 0.0001) on *Comt* expression in murine prefrontal cortex.

A combined administration of DPCPX and D–cycloserine significantly decreased *Comt* expression in murine prefrontal cortex compared to the saline- (*p* < 0.0001), DPCPX- (*p* < 0.0001) and D–cycloserine-treated group (*p* < 0.0001) (Fig. 3). Additionally, a single administration of D–cycloserine significantly increased *Comt* expression in murine prefrontal cortex when compared to the saline-treated group (*p* < 0.0001), and DPCPX blocked this effect and even reduced *Comt* expression below the value reported for the control group.

*DPCPX and MK–801 combination.* Two-way ANOVA indicated a significant effect of DPCPX (F_1,20_=25.87, *p* < 0.0001) and MK–801 (F_1,20_=98.66, *p* < 0.0001), and a significant DPCPX × MK–801 interaction (F_1,20_=14.39, *p* = 0.0011) on *Comt* expression in murine prefrontal cortex.

A combined administration of DPCPX and MK–801 significantly increased *Comt* expression in murine prefrontal cortex compared to the saline- (*p* < 0.0001), DPCPX- (*p* < 0.0001), and MK–801-treated group (*p* < 0.0001) (Fig. 3). Additionally, a single administration of MK–801 significantly increased *Comt* expression in murine prefrontal cortex when compared to the saline-treated group (*p* < 0.001), and DPCPX enhanced this effect.

*DPCPX and CGP 37849 combination.* Two-way ANOVA indicated a significant effect of DPCPX (F_1,20_=198.4, *p* < 0.0001) and CGP 37849 (F_1,20_=267.7, *p* < 0.0001), and a significant DPCPX × CGP 37849 interaction (F_1,20_=146.3, *p* < 0.0001) on *Comt* expression in murine prefrontal cortex.

A combined administration of DPCPX and CGP 37849 significantly increased *Comt* expression in murine prefrontal cortex compared to the saline- (*p* < 0.0001), DPCPX- (*p* < 0.0001), and CGP 37849-treated group (*p* < 0.0001) (Fig. 3). Additionally, a single administration of CGP 37849 significantly increased *Comt* expression in murine prefrontal cortex when compared to the saline-treated group (*p* < 0.05), and DPCPX enhanced this effect.

*Istradefylline and L–701*,*324 combination.* Two-way ANOVA indicated a significant effect of istradefylline (F_1,20_=364.3, *p* < 0.0001) and L–701,324 (F_1,20_=589.9, *p* < 0.0001), and a significant istradefylline × L–701,324 interaction (F_1,20_=807.1, *p* < 0.0001) on *Comt* expression in murine prefrontal cortex.

A combined administration of istradefylline and L–701,324 significantly decreased *Comt* expression in murine prefrontal cortex compared to the saline- (*p* < 0.0001), istradefylline- (*p* < 0.0001), and L–701,324-treated group (*p* < 0.0001) (Fig. 3).

*Istradefylline and D–cycloserine combination.* Two-way ANOVA indicated no effect of istradefylline (F_1,20_=1.781, *p* = 0.1970) and a significant effect of D–cycloserine F_1,20_=93.01, *p* < 0.0001), and no significant istradefylline × D–cycloserine interaction (F_1,20_=1.808, *p* = 0.1938) on *Comt* expression in murine prefrontal cortex (Fig. 3).

*Istradefylline and MK–801 combination.* Two-way ANOVA indicated a significant effect of istradefylline (F_1,20_=27.37, *p* < 0.0001) and MK–801 (F_1,20_=45.58, *p* < 0.0001), and a significant istradefylline × MK–801 interaction (F_1,20_=120.9, *p* < 0.0001) on *Comt* expression in murine prefrontal cortex.

A combined administration of istradefylline and MK–801 significantly decreased *Comt* expression in murine prefrontal cortex compared to the MK–801- (*p* < 0.0001) and istradefylline-treated group (*p* < 0.05) (Fig. 3). Additionally, a single administration of MK–801 significantly increased *Comt* expression in murine prefrontal cortex when compared to the saline-treated group (*p* < 0.001), and istradefylline blocked this effect.

*Istradefylline and CGP 37849 combination.* Two-way ANOVA indicated a significant effect of istradefylline (F_1,20_=213.3, *p* < 0.0001) and CGP 37849 (F_1,20_=134.1, *p* < 0.0001), and a significant istradefylline × CGP 37849 interaction (F_1,20_=500.6, *p* < 0.0001) on *Comt* expression in murine prefrontal cortex.

A combined administration of istradefylline and CGP 37849 significantly decreased *Comt* expression in murine prefrontal cortex compared to the saline- (*p* < 0.0001), istradefylline- (*p* < 0.0001), and CGP 37849-treated group (*p* < 0.0001) (Fig. 3). Additionally, a single administration of CGP 37849 significantly increased *Comt* expression in murine prefrontal cortex when compared to the saline-treated group (*p* < 0.05), and istradefylline blocked this effect and even reduced *Comt* expression below the value reported for the control group.

#### Slc6a15 gene expression

*DPCPX and L–701*,*324 combination.* Two-way ANOVA indicated a significant effect of DPCPX (F_1,20_=10.39, *p* = 0.0043) and no significant effect of L–701,324 (F_1,20_=1.614, *p* = 0.2185), and a significant DPCPX × L–701,324 interaction (F_1,20_=6.575, *p* = 0.0185) on *Slc6a15* expression in murine prefrontal cortex.

A combined administration of DPCPX and L–701,324 significantly decreased *Slc6a15* expression in murine prefrontal cortex compared to the saline- (*p* < 0.001), DPCPX- (*p* < 0.05), and L–701,324-treated group (*p* < 0.01) (Fig. 3).

*DPCPX and D–cycloserine combination.* Two-way ANOVA indicated a significant effect of DPCPX (F_1,20_=7.724, *p* = 0.0116) and D–cycloserine (F_1,20_=95.29, *p* < 0.0001), and no significant DPCPX × D–cycloserine interaction (F_1,20_=2.938, *p* = 0.1020) on *Slc6a15* expression in murine prefrontal cortex (Fig. 3).

*DPCPX and MK–801 combination.* Two-way ANOVA indicated a significant effect of DPCPX (F_1,20_=8.191, *p* = 0.0096) and MK–801 (F_1,20_=29.34, *p* < 0.0001), and a significant DPCPX × MK–801 interaction (F_1,20_=10.91, *p* = 0.0036) on *Slc6a15* expression in murine prefrontal cortex.

A combined administration of DPCPX and MK–801 significantly increased *Slc6a15* expression in murine prefrontal cortex compared to the saline- (*p* < 0.001), DPCPX- (*p* < 0.0001), and MK–801-treated group (*p* < 0.001) (Fig. 3).

*DPCPX and CGP 37849 combination.* Two-way ANOVA indicated a significant effect of DPCPX (F_1,20_=111.2, *p* < 0.0001) and CGP 37849 (F_1,20_=130.9, *p* < 0.0001), and a significant DPCPX × CGP 37849 interaction (F_1,20_=130.1, *p* < 0.0001) on *Slc6a15* expression in murine prefrontal cortex.

A combined administration of DPCPX and CGP 37849 significantly increased *Slc6a15* expression in murine prefrontal cortex compared to the saline- (*p* < 0.0001), DPCPX- (*p* < 0.0001), and CGP 37849-treated group (*p* < 0.0001) (Fig. 3).

*Istradefylline and L–701*,*324 combination.* Two-way ANOVA indicated a significant effect of istradefylline (F_1,20_=44.10, *p* < 0.0001) and L–701,324 (F_1,20_=58.06, *p* < 0.0001), and a significant istradefylline × L–701,324 interaction (F_1,20_=37.40, *p* < 0.0001) on *Slc6a15* expression in murine prefrontal cortex.

A combined administration of istradefylline and L–701,324 significantly increased *Slc6a15* expression in murine prefrontal cortex compared to the istradefylline-treated group (*p* < 0.0001) (Fig. 3).

*Istradefylline and D–cycloserine combination.* Two-way ANOVA indicated a significant effect of istradefylline (F_1,20_=32.73, *p* < 0.0001) and D–cycloserine (F_1,20_=95.40, *p* < 0.0001), and a significant istradefylline × D–cycloserine interaction (F_1,20_=172.3, *p* < 0.0001) on *Slc6a15* expression in murine prefrontal cortex.

A combined administration of istradefylline and D–cycloserine significantly increased *Slc6a15* expression in murine prefrontal cortex compared to the saline- (*p* < 0.0001), istradefylline- (*p* < 0.0001), and D–cycloserine-treated group (*p* < 0.0001) (Fig. 3). Additionally, a single administration of D–cycloserine significantly decreased *Slc6a15* expression in murine prefrontal cortex when compared to the saline-treated group (*p* < 0.0001), and istradefylline blocked this effect and even enhanced *Slc6a15* expression above the value reported for the control group.

*Istradefylline and MK–801 combination.* Two-way ANOVA indicated a significant effect of istradefylline (F_1,20_=8.699, *p* = 0.0079) and MK–801 (F_1,20_=199.5, *p* < 0.0001), and a significant istradefylline × MK–801 interaction (F_1,20_=135.1, *p* < 0.0001) on *Slc6a15* expression in murine prefrontal cortex.

A combined administration of istradefylline and MK–801 significantly increased *Slc6a15* expression in murine prefrontal cortex compared to the saline- (*p* < 0.0001), istradefylline- (*p* < 0.0001), and MK–801-treated group (*p* < 0.0001) (Fig. 3).

*Istradefylline and CGP 37849 combination.* Two-way ANOVA indicated a significant effect of istradefylline (F_1,20_=56.37, *p* < 0.0001) and CGP 37849 (F_1,20_=133.7, *p* < 0.0001), and a significant istradefylline × CGP 37849 interaction (F_1,20_=132.6, *p* < 0.0001) on *Slc6a15* expression in murine prefrontal cortex.

A combined administration of istradefylline and CGP 37849 significantly increased *Slc6a15* expression in murine prefrontal cortex compared to the saline- (*p* < 0.01), istradefylline- (*p* < 0.0001), and CGP 37849-treated group (*p* < 0.05) (Fig. 3).

## Discussion

It was found out that the selective antagonism of A1 adenosine receptors (by DPCPX [39, 40]) or A2A adenosine receptors (by istradefylline [39, 40]) in combination with modulation of NMDA receptors (by L–701,324, CGP 37849, and MK–801) reduced the duration of immobility of animals in the FST, which indicated the antidepressant effect. In addition, the antidepressant effect in mice was stronger after the use of NMDA receptor ligands with istradefylline than after their administration in combination with DPCPX. The antidepressant potential of combinations of subeffective doses of caffeine (a non-selective adenosine receptor antagonist, 5 mg/kg, *ip*) and the NMDA receptor ligands was also demonstrated in our previous study [35]. A significant reduction was observed in the immobility time in the FST in mice when animals received caffeine with L–701,324 (1 mg/kg, *ip*), MK–801 (0.05 mg/kg, *ip*), CGP 37849 (0.3 mg/kg, *ip*) and D–cycloserine (2.5 mg/kg, *ip*), which indicated the synergy of the tested combinations [35]. In present research, istradefylline, but not DPCPX, potentiated the antidepressant activity of D–cycloserine in the applied behavioral test. These inconsistencies may result from differences in the mechanism underlying the action of DPCPX and istradefylline. After the selective inhibition of A1 adenosine receptors by DPCPX, activation of Ca^2+^ voltage-dependent channels and blockage of K^+^ channels take place, which lead to the activation of adenylyl cyclase and weakening of cell membranes hyperpolarization. Consequently, an increase in the levels of noradrenaline, dopamine, and serotonin in the CNS is observed [28, 41]. In turn, the selective inhibition of A2A adenosine receptors by istradefylline contributes to the reduction of concentrations of these monoaminergic neurotransmitters (i.e., noradrenaline, dopamine, and serotonin) in the CNS, or it does not cause any changes in their levels [25]. As for the effect of selective antagonists of A1 and A2A adenosine receptors on glutamatergic transmission, stimulation, or reduction of Glu release in the brain is regarded, respectively [22–24, 42–44].

The paradoxical observation that both adenosine A1 and A2A receptor antagonists reduce immobility time in the FST, despite their opposing effects on glutamatergic neurotransmission, suggests a complex interplay between the modulatory role of adenosine receptors and the resulting behavioral outcomes. Several hypotheses can be put forward to explain these findings.

Attenuation of depressive behavior in mice observed in our research, resulting from the synergism of selected NMDA receptor ligands and istradefylline, and to a lesser extent DPCPX, might be explained by its impact on the heterotetrameric complexes of A1-A2A adenosine receptors located on the presynaptic glutamatergic nerve endings in the striatum [19, 20]. In a study by Ciruela et al. [20], coimmunoprecipitation, bioluminescence, and time-resolved fluorescence resonance energy transfer techniques were employed to demonstrate the presence of A1-A2A heteromers on the surface of cotransfected cells. Moreover, the same research team in in vivo studies carried out in the rat striatum established that selective activation of the A1 adenosine receptor in this receptor complex decreases glutaminergic neurotransmission, whereas A2A adenosine receptor stimulation inhibits the activity of A1 receptors and augments the release of Glu in the CNS [20]. Hence, selective A1 and A2A receptor antagonists via blocking the A1 and A2A receptors in the A1-A2A adenosine receptors complex could intensify or impair glutamatergic transmission in the striatum, respectively. Recent studies also suggested the presence of A2A-D2-NMDA receptors complex on GABAergic neurons in some parts of the CNS (mainly in globus pallidus and striatum) [45]. Selective blocking included in this complex A2A adenosine receptor by istradefylline administration could activate the D2 receptor and consequently might inhibit the function of the NMDA receptor in the A2A-D2-NMDA receptors complex. Therefore, the stronger antidepressant effect in the mice FST in the group treated with istradefylline with ligands modulating the activity of the NMDA receptors is probably the result of their synergistic inhibitory effect on glutamatergic neurotransmission. However, in the case of combinations of DPCPX with the NMDA receptor ligands, weaker antidepressant activity or even no antidepressant activity might result from divergent mechanisms of their action. Moreover, these differences could also be a consequence of receptor-receptor interactions in mentioned above heteroreceptor complexes.

The CNS is characterized by an intricate balance of excitatory and inhibitory neurotransmission. The opposing actions on Glu release by A1 and A2A receptor antagonists may trigger homeostatic compensatory responses. These compensations could normalise neurotransmission or activate alternative pathways that ultimately lead to a reduction in immobility time of mice in the FST. For example, inhibition of A1 receptors would typically enhance Glu release, but this might be balanced by increased GABAergic activity or by activation of downstream signaling pathways that moderate the increased excitatory transmission (for review see [46]). Additionally, glutamatergic neurotransmission does not operate in isolation but is modulated by and interacts with various neurotransmitter systems, such as dopamine, serotonin, and norepinephrine [47], all of which have been linked to the pathophysiology of depression and the response to antidepressants [48]. The effects of adenosine receptor antagonism on these systems may contribute to the observed behavioral changes and could provide an additional layer of complexity. Moreover, the FST encompasses behavioral despair and coping strategies, which are influenced by cognitive processes [49]. The adenosine receptor antagonists may be affecting these cognitive aspects of behavior in the FST [50], which may not directly correlate with changes in glutamatergic neurotransmission. However, it should be noted that this is a preliminary study. The discussed potential mechanisms of action of drugs on the release of neurotransmitters in this context are speculative, especially in relation to the literature findings that indicate an inverse effect of A2A and A1 antagonists on DA, 5-HT, NA and Glu levels. To further elucidate these mechanisms, additional studies employing a combination of pharmacological interventions, genetic models, and advanced imaging techniques are necessary. Moreover, to quantify neurotransmitters in brain structures, it would be worth using the brain microdialysis technique. These studies should aim to dissect the contribution of each receptor subtype in different brain regions and their impact on the complex behavioral phenotypes associated with depression and stress.

Several studies show that the level of peripheral BDNF in the serum could be considered as a biomarker, inter alia, of depressive symptoms severity [33, 34]. Since the concentration of this neurotrophic factor in the brain of patients suffering from depression is possible to assess only *post-mortem*, a diagnostic value has the BDNF level in blood [33, 34, 51]. Preclinical research has demonstrated that BDNF is capable of crossing the blood-brain barrier in both directions, thereby establishing a relationship between its central and peripheral levels [52, 53]. Moreover, Klein et al. [52] demonstrated that the serum BDNF level correlates positively with its level both in the frontal cortex and hippocampus. Therefore, it was resolved to ascertain the BDNF concentration in the murine serum.

Concerning the FST results, the outcomes of biochemical examination are surprising, because the vast majority of agents with a documented antidepressant activity, both in clinical and preclinical studies, lead to BDNF level enhancement in human and animal serum and/or brain tissue (for review see [54]). It is also important to note that clinical studies have demonstrated a reduction in serum BDNF levels in individuals diagnosed with major depressive disorder and following the administration of antidepressant treatment, these levels have been observed to normalise.

In the present studies, an increase in the concentration of this biomarker was observed only in group that received D–cycloserine when compared to the control group. Increase in BDNF level in murine serum observed in group administered with combination of istradefylline with D–cycloserine resulted from the own stimulatory effect of D–cycloserine on BDNF level. Likely, an acute administration of both selective A1 and A2A adenosine receptor inhibitors, as well as NMDA receptor ligands at per se subactive doses and their combinations, is insufficient to develop BDNF up-regulation. The more so that literature data point out that such changes appear mainly after several days of antidepressant administration (for review see [54]). Perhaps the outcomes obtained in our study result from the fact that in mice exposed to acute environmental stress, such as the FST, there was no reduction in serum BDNF levels, and therefore it was not necessary to normalise this parameter. This requires further study in animal models of major depression after long-term drug administration.

Finally, the mRNA level of genes (i.e. *Adora1*, *Comt*, and *Slc6a15*) in the murine prefrontal cortex, which may play a role in the pathophysiology and/or pharmacotherapy of depressive disorder was examined. In conducting our research, we selected the prefrontal cortex as the region of interest due to its consistent impairment in major depressive disorder. Stress, especially strong and/or chronic, causes numerous changes in animal behavior. Research has shown that these behaviors are accompanied by structural and functional alterations in the prefrontal cortex, analogous to those documented in patients suffering from major depressive disorder [55].

The adenosine A1 receptor (*Adora1*) represents one of three adenosine receptor types. It belongs to the G protein-coupled receptor 1 family, and its endogenous ligand is adenosine [56]. These receptors are expressed in the CNS (especially in hippocampus, cerebral cortex, cerebellum, brain stem, and thalamus), where they are responsible for the modulation and release of neurotransmitters, as well as the plasticity of neurons [40]. There is research that shows the potential connection between improving the A1 receptor signaling and the antidepressant effect on people sufferring from depressive disorder. This, in turn, indicates that some disturbances associated with the A1 receptor may play a role in the pathophysiology of the depression [56]. In addition, they play a neuroprotective role in psychiatric diseases, i.e. depression, bipolar disorders, anxiety (for review see [39, 40]).

As observed before [57], an increase in the level of *Adora1* in the murine prefrontal cortex may be induced by caffeine (10 mg/kg/day for 14 days). Based on the evaluation of the relative expression of the *Adora1* gene conducted in our study, it was shown that both DPCPX and istradefylline at the subactive doses do not result in significant alterations in the mRNA expression of this gene in the examined CNS structure. It is probable that in the treatment regimen (single administration, subtherapeutic dose) applied in our experiments, the chosen selective A1 and A2A adenosine receptor antagonists were not able to influence the mRNA level of *Adora1*. Likewise, in our earlier studies [32], neither DPCPX nor istradefylline caused changes in the expression of the gene encoding A1 adenosine receptors. In turn, the administration of L–701,324 in monotherapy and DPCPX in combination with D–cycloserine and CGP 37849 resulted in an increase in *Adora1* expression compared to the control group. Similarly, Szopa et al. [32] also observed changes in *Adora1* expression in mice that received jointly DPCPX and ionic ligands of the NMDA receptor. DPCPX + Mg^2+^ caused a reduction, whereas DPCPX + Zn^2+^ an increase in the mRNA level of this gene. The observed enhancement in the expression of this gene may be attributed to the elevation of neurotransmitter concentration in the CNS of animals. As demonstrated by Serchov et al. [56], the increased expression of A1 adenosine receptors in the medial prefrontal cortex contributes to the reduction of depressive incidence in behavioral tests (FST and TST) in mice [56], and results in an antidepressant effect in an animal model of chronic stress [56, 58]. Moreover, non-pharmacological methods of treating depression, such as sleep deprivation or electroconvulsive therapy, have been proven to cause increased activation of the A1 adenosine receptors [59–61]. In addition, Hines et al. [61] and Serchov et al. [56] proved that enhanced transmission via adenosine A1 receptors resulting from the augmented expression of these receptors on the forebrain neurons of transgenic mice or from the administration of a selective A1 receptor agonist supports the reduction of depressive behavior. They also indicated that A1 receptors are necessary to induce the antidepressant effects of the above-mentioned non-pharmacological methods of treating depression [56, 61]. Interestingly, in the present experiment, in the case of mice receiving injections of D–cycloserine, MK–801, and CGP 37849 a decrease in *Adora1* expression in the mice prefrontal cortex was noted, however, the additional application of DPCPX, but not istradefylline, resulted in its restoration to a level like or even higher than measured in control animals. Similar outcomes were obtained by Mendiola-Precoma et al. [62], who observed that a non-selective adenosine receptor antagonist, theobromine (0.5 and 30 mg/l, *po*), restored or even increased mRNA levels of *Adora1* in a rat brain Alzheimer’s disease model. These surprising study results reveal a role for the *Adora1* gene in the effects of drugs administered to mice in our study, but further, more focused research is required to determine its exact contribution.

Catechol-*O*-methyltransferase (*Comt*) represents a crucial human enzyme involved in the breakdown of catecholamines [63]. The enzyme catalyses the transfer of methyl groups from *S*-adenosylmethionine to catecholamines (e.g., dopamine, norepinephrine, epinephrine). Described *O*-methylation contributes to some degenerative pathways of the catecholamine neurotransmitters. A number of studies have demonstrated a correlation between the *Comt* and a range of mental health conditions like obsessive-compulsive disorder, schizophrenia, anorexia nervosa or depressive disorder [64]. Potentially, the development of those disorders may be attributed to certain alterations of the *Comt* in the prefrontal cortex. To be more specific, modifications may be related to 22q11.2 deletion syndrome, which leads to disturbed regulation of this enzyme in the brain [63]. Because of (1) the well-documented status of *Comt* as a most important enzyme engaged in the degradation process of catecholamines (adrenaline, noradrenaline, and dopamine) [65], (2) positive correlation between depression and *COMT* gene (for review see [63]), and (3) evidence showing that chronic (21-day) treatment with fluoxetine causes down-regulation of *Comt* mRNA level in rat frontal cortex [66], the expression of *Comt* in the prefrontal cortex in mice was evaluated. A decrease in the mRNA level of *Comt* was detected in the brain tissue of animals receiving DPCPX with L–701,324 or istradefylline with L–701,324 or CGP 37849. Moreover, in the case of the combined administration of istradefylline with MK–801, normalization of the elevated mRNA levels of *Comt*, increased as a result of MK–801 administration, to the ones recorded in the saline-treated group was observed. According to the literature mentioned above, it can be assumed that this is a probable mechanism underlying compensatory changes occurring in the brain of rodents subjected to acute environmental stress such as the FST. In order to maintain levels of neurotransmitters in response to stressors, the expression of the gene encoding the enzyme that breaks them down is lowered. On the other hand, results obtained in the experimental groups receiving D–cycloserine, MK–801 or CGP 37849 seems to be surprising since the potentiation of *Comt* expression in the prefrontal cortex was detected. However, similar outcomes were demonstrated in our previous studies [32], in which a significant increase in *Comt* expression in mice receiving DPCPX or ionic NMDA receptor ligands, i.e., Mg^2+^ and Zn^2+^ was observed.

Our study revealed that the drug combination DPCPX + D–cycloserine increased *Adora1* and decreased *Comt* expression but did not reveal statistically significant changes in FST. This is a surprising observation because it appears that increased *Adora1* expression and decreased *Comt* expression may be associated with antidepressant effects. Nevertheless, it is important to acknowledge that behavioral tests have certain limitations. Animals’ behavior during forced swimming may be influenced by many external factors and individual characteristics of rodents. The interpretation of the period of stillness also presents some difficulties. Regarding the results of molecular tests, which are free from such limitations, it seems advisable to investigate the antidepressant effect of the drug combination DPCPX + D–cycloserine in other behavioral tests and the effects of chronic drug treatment should also be studied.

Solute carrier family 6 member 15 (SLC6a15) is a sodium-dependent transporter responsible for the uptake and release of neutral amino acids. It is connected especially with the transport of leucine, valine, isoleucine, and methionine [67]. There is a probability, that this gene may be linked to the onset of major depression. The research shows a correlation between chronic stress in mice and a reduction in the *Slc6a15* in the hippocampus. It is plausible that these alterations may give rise to disturbances in neuronal conduction, thus rendering the *Slc6a15* protein a promising target for antidepressant therapies [67, 68]. Recent data presented that *Slc6a15* knockout rodents exhibit reduced depressive and anxiety behavior [69–71], whereas the overexpression of *Slc6a15* in the hippocampus resulted in mood disorders [69]. In the present research, mice receiving a single dose of D–cycloserine, as well as DPCPX together with L–701,324 reduced mRNA levels of *Slc6a15* in the prefrontal cortex when compared to the control group. These results indicate a possible role of *Slc6a15* in manifesting the antidepressant effect of the above-mentioned substances. On the other hand, an addition of DPCPX to the treatment with CGP 37849 or MK–801, as well as an addition of istradefylline to the therapy with D–cycloserine or MK–801 resulted in an increase in the mRNA level of *Slc6a15* in the murine prefrontal cortex to a level much higher than in the control group. Similar outcomes were obtained when DPCPX or istradefylline was co-administered with Zn^2+^ [32].

The outcomes of both biochemical and molecular studies are inconclusive. It is difficult to explain the mechanisms involved in changes in BDNF levels and expression of *Adora1*, *Comt*, and *Slc6a15* observed in our study. Perhaps one of the reasons for the described inconsistencies is a complex mechanism of interaction between the adenosinergic and glutamatergic systems in the CNS. Therefore, further experiments, including chronic studies, are needed.

To sum up, the present results indicate a positive interaction between selective antagonists of A1 and A2A adenosine receptors with NMDA receptor ligands, which was manifested as an antidepressant effect in the FST. Our results may serve as a confirmation of the existence of an interaction between the adenosinergic system and the glutamatergic system. Nevertheless, the results shown in this paper point out that the simultaneous administration of subeffective doses of selective A1 or A2A adenosine receptor antagonists with subeffective doses of NMDA receptor ligands may be considered a new, more effective, and safer strategy for the treatment of patients suffering from depressive disorders. The outcomes of our experiments, however, cannot be directly applied to clinical practice but may be an inspiration to undertake further research related to the role of the interaction between adenosinergic and glutamatergic systems as a mechanism underlying the pathogenesis and therapy of depression.

## Limitations of the study

The main limitations of the current study are: (1) the application of a single-administration regimen of tested combinations of selective A1 and A2A adenosine receptor antagonists with NMDA receptor ligands, (2) the lack of confirmation of the results obtained in the FST in another test commonly used to study depression, e.g. tail suspension test (TST), (3) the lack of experiments assessing the brain levels of neurotransmitters involved in the development of depression, and (4) the BDNF levels were detected in serum, which provided an indirect measure of BDNF levels in the brain. It should be emphasized that due to the above-mentioned limitations of our studies, the results should be treated as preliminary and require confirmation.

## Data Availability

The data used in this study are available from the corresponding author upon reasonable request.

## Abbreviations

*Adora1*: Adenosine A1 receptor gene
BDNF: Brain-derived neurotrophic factor
CNS: Central nervous system
*Comt*: Catechol–*O*–methyl–transferase gene
FST: Forced swim test
Glu: Glutamate
*ip*: Intraperitoneally
NMDA: *N*-methyl-D-aspartate
*po*: Per os (by oral gavage)
*Slc6a15*: Solute carrier family 6 (neurotransmitter transporter) member 15 genes
